# Supplementary material for: Heat adaptation of phage T7 under an extended genetic code
Source: Virus Evol. 2021 Dec 1;7(2):veab100. doi: 10.1093/ve/veab100 (PMC8923235; doi:10.1093/ve/veab100)
Supplement: veab100_Supp [file veab100_supp.zip › Sequencing_Analysis.zip!/Sequencing_Analysis/S2/evidence/MC_SIDE_2_39.html]

BRESEQ :: Evidence


| Predicted mutation | | | | | | |
| --- | --- | --- | --- | --- | --- | --- |
| evidence | seq id | position | mutation | annotation | gene | description |
| MC JC | NC\_001604.1 | 13,579 | Δ769 bp | intergenic (–/–) | – / – | –/– |

| Missing coverage evidence... | | | | | | | | | | |
| --- | --- | --- | --- | --- | --- | --- | --- | --- | --- | --- |
|  |  |  | seq id | start | end | size | ←reads | reads→ | gene | description |
| \* | \* | ÷ | NC\_001604.1 | 13579 | 14347 | 769 | 5494 [43] | [34] 5482 | *–/–* | –/– |

| New junction evidence | | | | | | | | | | | |
| --- | --- | --- | --- | --- | --- | --- | --- | --- | --- | --- | --- |
|  | | seq id | position | reads (cov) | reads (cov) | score | skew | freq | annotation | gene | product |
| \* | ? | NC\_001604.1 | = 13578 | 43 (0.010) | 5271 (1.020) | 434/584 | 0.0 | 99.3% | intergenic (–/–) | *–/–* | –/– |
|  | ? | NC\_001604.1 | 14348 = | 29 (0.010) |  |  |  |  | intergenic (–/–) | *–/–* | –/– |

Only 100 of 5482 total aligned reads displayed.  

`AAGACAGACTGGCTGGAAGAAACCAGAAGTTACCAAGCTGAGTCATAAGGTGTTCGATAAGGACACTATGACCCACATCAAGGCTGGTGATTGGGTTAAGGTTGACATGGGAGTTGTTGGTGGATACGGCTACGTCCGCTCAGTTAGTGGCAAATATGCACAAGTGTCATACATCACAGGTGTTACTCCACGCGGTGCAATCGTTGCCGATAAGACCAACATGATTCACACAGGTTTCTTGACAGTTGTTTCATATGAAGAGATTGTTAAGTCACGATAATCAATAGGAGAAATCAATATGATCGTTTCTGACATCGAAGCTAACGCCCTCTTAGAGAGCGTCACTAAGTTCCACTGCGGGGTTATCTACGACTACTCCACCGCTGAGTACGTAAGCTACCGTCCGAGTGACTTCGGTGCGTATCTGGATGCGCTGGAAGCCGAGGTTGCACGAGGCGGTCTTATTGTGTTCCACAACGGTCACAAGTATGACGTTCCTGCATTGACCAAACTGGCAAAGTTGCAATTGAACCGAGAGTTCCACCTTCCTCGTGAGAACTGTATTGACACCCTTGTGTTGTCACGTTTGATTC  >  NC_001604.1/14055‑14647`  

                                                                                                                                                                                                                                                                                                     `|`                                                                                                                                                                                                                                                                                                             
`cagacgactacgagaagtttcgctggagagtcccattctaatacgactcactaaaggagacacaccatgttcaaactgattaagaagttaggccaactgctggttcgtatgtacaacgtggaagccaagcgactgaacgatgaggctcgtaaagaggccacacagtcacgcgctctggcgatttgctccaacgaactggctgacagtgcatccactaaagttaccgaggctgcccgtgtggcaaaccaagctcaacagctttccaaattctttgagtaatcaaacaggagaaaTCAAGATg                                                                                                                                                                                                                                                                                                      <  1:34649‑M2/8‑1 (MQ=255)`  
       `ctacgagaagtttcgctggagagtcccattctaatacgactcactaaaggagacacaccatgttcaaactgattaagaagttaggccaactgctggttcgtatgtacaacgtggaagccaagcgactgaacgatgaggctcgtaaagaggccacacagtcacgcgctctggcgattcgctccaacgaactggctgacagtgcatccactaaagttaccgaggctgcccgtgtggcaaaccaagctcaacagctttccaaattctttgagtaatcaaacaggagaaatcctgtctcttatac                                                                                                                                                                                                                                                                                               >  1:121612‑M2/287‑288 (MQ=255)`  
        `ataaggtccagtcgctggagagtcccattctaatactactcactaacggatacacaccatgttcaaactgattaagaagttaggccaactgctggttcgtatgtacaacgtggaatccaagcgactgaactatgaggctcgtaaagatgccacacagtcacgcgctctggcgattcgctccaacgaactggctgacagtgcatccactaaagttaccgaggctgcccgtgtggcaaaccaagctcaacagctttccaaattctttgagtaatcaaacaggagaaaTCAATATGATCGTTTc                                                                                                                                                                                                                                                                                              <  1:570094‑M2/16‑1 (MQ=255)`  
           `ctcggagatgtgtataagagacagttctaatacgactcactaaaggagacacaccatgttcaaactgattaagaagttaggccaactgctggttcgtatgtacaacgtggaagccaagcgactgaacgatgaggctcgtaaagaggccacacagtcacgcgctctggcgattcgctccaacgaactggctgacagtgcatccactaaagttaccgaggctgcccgtgtggcaaaccaagctcaacagctttccaaattctttgagtaatcaaacaggagaaaTCAATATGATCGTTTCTGa                                                                                                                                                                                                                                                                                           <  1:763187‑M2/19‑1 (MQ=255)`  
                    `tcgctggagagtcccattctaatacgactcactaaaggagacacaccatgttcaaactgattaagaagttaggccaactgctggttcgtatgtacaacgtggaagccaagcgactgaacgatgaggctcgtaaagaggccacacagtcacgcgctctggcgattcgctccaacgaactggctgacagtgcatccactaaagttaccgaggctgcccgtgtggcaaaccaagctcaacagctttccaaattctttgagtaataaataggagaaaTCAATATGATCGTTTCTGACATCGAAGc                                                                                                                                                                                                                                                                                  >  1:158541‑M2/274‑301 (MQ=255)`  
                     `gctggagagtcccattctaatacgactcactaaaggagacacaccatgttcaaactgattaagaagttaggtcaactgctggttcgtatgtacaacgtggaagccaagcgactgaacgatgaggctcgtaaagaggccacacagtcacgcgctctggcgattcgctccaacgaactggctgacagtgcatccactaaagttaccgaggctgcccgtgtggcaaaccaagctcaacagctttccaaattctttgagtaatcaaacaggagaaaTCAATATGATCGTTTCTGACATCGAAGCt                                                                                                                                                                                                                                                                                 <  1:669536‑M2/29‑1 (MQ=255)`  
                             `gtcccattctaatacgactcactaaaggagacacaccatgttcaaactgattaagaagttaggccaactgctggttcgtatgtacaacgtggaagccaagcgactgaacgatgaggctcgtaaagaggccacacagtcacgcgctctggcgattcgctccaacgaactggctgacagtgcatccactaaagttaccgaggctgcccgtgtggcaaaccaagctcaacagctttccaaattctttgagtaatcaaacaggagaaatcaatatctgtctcttatgcacatctccgagcccacg                                                                                                                                                                                                                                                                         >  1:403878‑M2/265‑271 (MQ=255)`  
                             `gtcccattctaatacgactcactaaaggagacacaccatgttcaaactgattaagaagttaggccaactgctggttcgtatgtacaacgtggaagccaagcgactgaacgatgaggctcgtaaagaggccacacagtcacgcgctctggcgattcgctccaacgaactggctgacagtgcatccactaaagttaccgaggatgcccgtgtggcaaaccaagctcaacagctttccaaattctttgagtaatcaaacaggagaaaCCATTATGATCGTTTCTGACATCGAAGCTAACGCCct                                                                                                                                                                                                                                                                         >  1:100118‑M2/265‑301 (MQ=255)`  
                                  `tgtataagagacagcactaaaggagacacaccatgttcaaactgattaagaagttaggccaactgctggttcgtatgtacaacgtggaagccaagcgactgaacgatgaggctcgtaaagaggccacacagtcacgcgctctggcgattcgctccaacgaactggctgacagtgcatccactaaagttaccgaggctgcccgtgtggcaaaccaagctcaacagctttccaaattctttgagtaatcaaacaggagaaaTCAATATGATCGTTTCTGACATCGAAGCTAACGCCCTCTTag                                                                                                                                                                                                                                                                    <  1:602644‑M2/42‑1 (MQ=255)`  
                                  `attctaatacgactcactaaaggagacacaccatgttcaaactgattaagaagttaggccaactgctggttcgtatgtacaacgtggaagccaagcgactgaacgatgaggctcgtaaagaggccacacagtcacgcgctctggcgattcgctccaacgaactggctgacagtgcatccactaaagttaccgaggctgcccgtgtggcaaaccaagctcaacagctttccaaattctttgagtaatcaaacaggagaaaTCAATATGATCGTTTCTGACATCGAAGCTAACGCCCTCTTag                                                                                                                                                                                                                                                                    >  1:749929‑M2/260‑301 (MQ=255)`  
                                  `attctaatacgactcactaaaggagacaaaccaggttcaaactgattaagaagttaggccaactgctggttcgtatgtacaacgtggaagccaagcgactgaacgatgaggctcgtaaagaggccacacagtcacgcgctctggcgattcgctccaacgaactggctgacagtgcatccactaaagttaccgaggctgcccgtgtggcaaaccaagctcaacagctttccaaattctttgagtaatcaaacaggagaaaTCAATATGATCGTTTCAGACATCGAAGCTAACACCCTCTTag                                                                                                                                                                                                                                                                    <  1:712680‑M2/42‑1 (MQ=255)`  
                                   `ttctaatacgactcactaaaggagacacaccatgttcaaactgattaagaagttaggccaactgctggttcgtatgtacaacgtggaagccaagcgactgaacgatgaggctcgtaaagaggccacacagtcacgcgctctggcgattcgctccaacgaactggctgacagtgcatccactaaagttaccgaggctgcccgtgtggcaaaccaagctcaacagctttccaaattctttgagtaatcaaataggagaaaTCAATATGATCGTTTCTGACATCGAAGCTAACGCCCTCTTaga                                                                                                                                                                                                                                                                   >  1:677204‑M2/259‑301 (MQ=255)`  
                                   `ttctaatacgactcactaaaggagacacaccatgttcaaactgattaagaagttaggccaactgctggttcgtatgtacaacgtggaagccaagcgactgaacgatgaggctcgtaaagaggccacacagtcacgcgctctggcgattcgctccaacgaactggctgacagtgcatccactaaagttaccgaggctgcccgtgtggcaaaccaagctcaacagctttccaaattctttgagtaatcaaacaggagaaaTCAATATGATCGTTTCTGACATCGAAGCTAACGCCCTCTTaga                                                                                                                                                                                                                                                                   >  1:742472‑M2/259‑301 (MQ=255)`  
                                   `ttctaatacgactcactaaaggagacacaccatgttcaaactgattaagaagttaggccaactgctggttcgtatgtacaacgtggaagccaagcgactgaacgatgaggctcgtaaagaggccacacagtcacgcgctctggcgattcgctccaacgaactggctgacagtgcatccactaaagttaccgaggctgcccgtgtggcaaaccaagctcaacagctttccaaattctctgagtaatcaaacaggggaaaTCAATATGATAGATTCTGACAGAGAAGCTAACGCCCTCTTaga                                                                                                                                                                                                                                                                   >  1:152075‑M2/259‑301 (MQ=255)`  
                                    `ttctaatacgactcactaaaggagacacaccatgttcaaactgattaagaagttaggccaactgctggttcgtatgtacaacgtggaagccaagcgactgaacgatgaggctcgtaaagaggccacacggtcacgcgctctggcgattcgctccaacgaactggctgacagtgcatccactaaagttaccgaggctgcccgtgtggcaaaccaagctcaacagctttccaaattctttgagtaatcaataggagaaaTCAATATGATCGTTTCTGACATCGAAGCTAACGCCCTCTTagag                                                                                                                                                                                                                                                                  >  1:72787‑M2/258‑301 (MQ=255)`  
                                    `tgtataagagacagctaaaggagacacaccatgttcaaactgattaagaagttaggccaactgctggttcgtatgtacaacgtggaagccaagcgactgaacgatgaggctcgtaaagaggccacacagtcacgcgctctggcgattcgctccaacgaactggctgacagtgcatccactaaagttaccgaggctgcccgtgtggcaaaccaagctcaacagctttccaaattctttgagtaatcaaacaggagaaaTCAATATGATCGTTTCTGACATCGAAGCTAACGCCCTCTTagag                                                                                                                                                                                                                                                                  <  1:282125‑M2/44‑1 (MQ=255)`  
                                    `tgtataagagacagctaaaggagacacaccatgttcaaactgattaagaagttaggccaactgctggttcgtatgtacaacgtggaagccaagcgactgaacgatgaggctcgtaaagaggccacacagtcacgcgctctggcgattcgctccaacgaactggctgacagtgcatccactaaagttaccgaggctgcccgtgtggcaaaccaagctcaacagctttccaaattctttgagtaatcaaacaggagaaaTCAATATGATCGTTTCTGACATCGAAGCTAACGCCCTCTTagag                                                                                                                                                                                                                                                                  <  1:556044‑M2/44‑1 (MQ=255)`  
                                    `tctaatacgactcactaaagtagacacaccatgttcaaactgattaagaagttaggccaactgctggttcgtatgtacaacgtggaagccaagcgactgaacgatgaggctcgtaaagaggccacacagtcacgcgctctggcgattcgctccaacgaactggctgacagtgcatccactaaagttaccgaggctgcccgtgtggcaaaccaagctcaacagctttccaaattctttgagtaatcaaacaggagaaaCCAATATGATCGTTTCTGACATCGAAGCTAACGCCCTCTTagag                                                                                                                                                                                                                                                                  <  1:421414‑M2/44‑1 (MQ=255)`  
                                    `tctaatacgactcactaaaggagacacaccatgttcaaactgattaagaagttaggccaactgctggttcgtatgtacaacgtggaagccaagcgactgaacgatgaggctcgtaaagaggccacacagtcacgcgctctggcgattcgctccaacgaactggctgacagtgcatccactaaagttaccgaggctgcccgtgtggcaaaccaagctcaacagctttccaaattctttgagtattcaaacaggagaaaTCAATATGATCGTTTCTGACATCGAAGCTAACGCCCTCTTagag                                                                                                                                                                                                                                                                  <  1:770662‑M2/44‑1 (MQ=255)`  
                                    `tctaatacgactcactaaaggagacacaccatgttcaaactgattaagaagttaggccaactgctggttcgtatgtacaacgtggaagccaagcgactgaacgatgaggctcgtaaagaggccacacagtcacgcgctctggcgattcgctccaacgaactggctgacagtgcatccactaaagttaccgaggctgcccgtgtggcaaaccaagctcaacagctttccaaattctttgagtaatcaaacaggagaaaTCAATATGATCGTTTCTGACATCGAAGCTAACGCCCTCTTagag                                                                                                                                                                                                                                                                  <  1:13619‑M2/44‑1 (MQ=255)`  
                                    `tctaatacgactcactaaaggagacacaccatgttcaaactgattaagaagttaggccaactgctggttcgtatgtacaacgtggaagccaagcgactgaacgatgaggctcgtaaagaggccacacagtcacgcgctctggcgattcgctccaacgaactggctgacagtgcatccactaaagttaccgaggctgcccgtgtggcaaaccaagctcaacagctttccaaattctttgagtaatcaaacaggagaaaTCAATATGATCGTTTCTGACATCGAAGCTAACGCCCTCTTagag                                                                                                                                                                                                                                                                  <  1:165436‑M2/44‑1 (MQ=255)`  
                                    `tctaatacgactcactaaaggagacacaccatgttcaaactgattaagaagttaggccaactgctggttcgtatgtacaacgtggaagccaagcgactgaacgatgaggctcgtaaagaggccacacagtcacgcgctctggcgattcgctccaacgaactggctgacagtgcatccactaaagttaccgaggctgcccgtgtggcaaaccaagctcaacagctttccaaattctttgagtaatcaaacaggagaaaTCAATATGATCGTTTCTGACATCGAAGCTAACGCCCTCTTagag                                                                                                                                                                                                                                                                  <  1:36313‑M2/44‑1 (MQ=255)`  
                                    `tataagagacagcactaaaggagaaacaccatgttcaaactgataaagaagttaggccaactgctggttcgtatgtacaacgtggaagccaagcgactgaacgatgaggctcgtaaagaggccacacagtcacgcgctctggcgattcgctccaacgaactggctgacagtgcatccacaaaagttaccgaggctgcccgtgtggcaaaccaagctcaacagctttccaaattctttgagtaatcaaacaggagaaaTCAATATGATCGTTTCTGACATCGAAGCTAACGCCCTCTTagag                                                                                                                                                                                                                                                                  <  1:784956‑M2/44‑1 (MQ=255)`  
                                    `cctaatacgactcactaaaggagacacaccatgttcgaactgattaagaagttaggccaactgatggttcgtatgtacaacgtggaagccaagcgactgaacgatgaggctcgtaaagaggccacacagtcacgcgctctggcgattcgctccaacgaactggctgacagtgcatccactaaagttaccgaggctgcccgtgtggcaaaccaagctcaacagctttccaaattctttgagtaatcaaacaggagaaaCCATTATGATCGTTTCTGACATCGAAGCTAACGCCCTCTTagag                                                                                                                                                                                                                                                                  <  1:436984‑M2/44‑1 (MQ=255)`  
                                    `acagatacgactcactaaaggagacaaaccatgttcaaactgattaagaagttaggccaactgctggttcgtatgtacaacgtggaagccaagcgactgaacgatgaggctcgtaaagaggccacacagtcacgcgctctggcgattcgctccaacgaactggctgacagtgcatccactaaagttaccgaggctgcccgtgtggcaaaccaagctcaacagctttccaaattctttgagtaatcaaacaggagaaaTCAATATGATCGTTTCTGACATCGAAGCTAACGCCCTCTTagag                                                                                                                                                                                                                                                                  <  1:395530‑M2/44‑1 (MQ=255)`  
                                      `gatgtgtataagagacaggagacacaccatgttcaaactgattaagaagttaggccaactgctggttcgtatgtacaacgtggaagccaagcgactgaacgatgaggctcgtaaagaggccacacagtcacgcgctctggcgattcgctccaacgaactggctgacagtgcatccactaaagttaccgaggctgcccgtgtggcaaaccaagctcaacagctttccaaattctttgagtaatcaaacaggagaaaTCAATATGATCGTTTCTGACATCGAAGCTAACGCCCTCTTagagag                                                                                                                                                                                                                                                                <  1:697807‑M2/46‑1 (MQ=255)`  
                                              `ctcactaaatgagacacaccatgttcaaactgattaagaagttaggccaactgctggttcgtatgtacaacgtggaagccaagcgactgaacgatgaggctcgtaaagaggccacacagtcacgcgcgatggcgattcgcgccaacgaactggcggaaagtgcatccactaaagttaccgaggcggcccgtgtggcaaaccaagctcaacagctttccaaatgctttgagtaatcaaacaggagaaaCCATTATGATCGTTTCTGACATCGAAGCTAACGCCCTCTTAGAGAGCGTCACTa                                                                                                                                                                                                                                                        <  1:65342‑M2/54‑1 (MQ=255)`  
                                              `agcactaaaggagacacaccatgttcaaactgattaagaagttaggccaactgctggttcgtatgtacaacgtggaagccaagcgactgaacgatgaggctcgtaaagaggccacacagtcacgcgctctggcgattcgctccaacgaactggctgacagtgcatccactaaagttaccgaggctgcccgtgtggcaaaccaagctcaacagctttccaaattctttgagtaatcaaacaggagaaaTCAATATGATCGTTTCTGACATCGAAGCTAACGCCCTCTTAGAGAGCGTCACTa                                                                                                                                                                                                                                                        <  1:79232‑M2/54‑1 (MQ=255)`  
                                                `cactaaaggagacacaccatgttcaaactgattaagaagttaggccaactgctggttcgtatgtacaacgtggaagccaagcgactgaacgatgaggctcgtaaagaggccacacagtcacgcgctctggcgattcgctccaacgaactggctgacagtgcatccactaaagttaccgaggctgcccgtgtggcaaaccaagctcaacagctttccaaattctttgagtaatcaaacaggagaaaTCAATATGATCGTTTCTGACATCGAAGCTAACGCCCTCTTAGAGAGCGTCCctact                                                                                                                                                                                                                                                      >  1:756117‑M2/246‑299 (MQ=255)`  
                                                `cactaaaggagacacaccatgttcaaactgattaagaagttaggccaactgctggttcgtatgtacaacgtggaagccaagcgactgaacgatgaggctcgtaaagaggccacacagtcacgcgctctggcgattcgctccaacgaactggctgacagtgcatccactaaagttaccgaggctgcccgtgtggcaaaccaagctcaacagctttccaaattctttgagtaatcaaacaggagaaaTCAATATGATCGTTTCTGACATCGAAGCTAACGCCCTCTTAGAGAGCGTCACTAAg                                                                                                                                                                                                                                                      >  1:643790‑M2/246‑301 (MQ=255)`  
                                                 `gagacaggagacacaccatgttcaaactgattaagaagttaggccaactgctggttcgtatgtacaacgtggaagccaagcgactgaacgatgaggctcgtaaagaggccacacagtcacgcgctctggcgattcgctccaacgaactggctgacagtgcatccactaaagttaccgaggctgcccgtgtggcaaaccaagctcaacagctttccaaattctttgagtaatcaaacaggagaaaTCAATATGATCGTTTCTGACATCGAAGCTAACGCCCTCTTAGAGAGCGTCACTAAGt                                                                                                                                                                                                                                                     <  1:143214‑M2/57‑1 (MQ=255)`  
                                                  `ctaaaggagacacaccatgttcaaactgattaagaagttaggccaactgctggttcgtatgtacaacgtggaagccaagcgactgaacgatgaggctcgtaaagaggccacacagtcacgcgctctggcgattcgctccaacgaactggctgacagtgcatccactaaagttaccgaggctgcccgtgtggcaaaccaagctcaacagctttccaaattctttgagtaatcaaacaggagaaaTCAATATGATCGTTTCTGACATCGAAGCTAACGCCCTCTTAGAGAGCGTCACTAAGtt                                                                                                                                                                                                                                                    >  1:229644‑M2/244‑301 (MQ=255)`  
                                                  `ctaaaggagacacaccatgttcaaactgattaagaagttaggccaactgctggttcgtatgtacaacgtggaagccaagcgactgaacgatgaggctcgtaaagaggccacacagtcacgcgctctggcgattcgctccaacgaactggctgacagtgcatccactaaagttaccgaggctgcccgtgtggcaaaccaagctcaacagctttccaaattctttgagtaatcaaacaggagaaaTCAATATGATCGTTTCTGACATCGAAGCTAACGCCCTCTTAGAGAGCGTAACTAAGtt                                                                                                                                                                                                                                                    >  1:564081‑M2/244‑301 (MQ=255)`  
                                                    `aaaggagacacaccatgttcaaactgattaagaagttaggccaactgctggttcgtatgtacaacgtggaagccaagcgactggacgatgaggctcgtaaagaggccacacagtcacgcgctctggcgattcgctccaacgaactggctgacagtgcatccactaaagttaccgaggctgcccgtgtggcaaaccaagctcaacagctttccaaattctttgagtaatcaaacaggagaaaTCAATATGATCGTTTCTGACATCGAAGCTACCGCCCTCTTAGAGAGCGTCACTAAGTTcc                                                                                                                                                                                                                                                  >  1:244321‑M2/242‑301 (MQ=255)`  
                                                      `ggggggctcggaaatgtgtataagagacaggaatttaggccaaatgatggttcgtatgtaaaacggggaagacaagcgactgaacgaggaggctcgtaaagaggccacacagtcacgcgatctggcgattcgctccaacgaactggctgacagtgcatccactaaagttaccgaggctgcccgggtggcaaaccaagctcaacagctttccaaattctttgagtaatcaataggagaaaTCAATATGATCGTTTCTGACATCGAAGCTAACGCCCTCTTAGAGAGCGTCACTAAGTTCCAc                                                                                                                                                                                                                                                <  1:483221‑M2/62‑1 (MQ=255)`  
                                                          `gacacaccatgttcaaactgattaagaagttaggccaactgctggttcgtatgtacaacgtggaagccaagcgactgaacgatgaggctcgtaaagaggccacacagtcacgcgctctggcgattcgctccaacgaactggctgacagtgcatccactaaagttaccgaggctgcccgtgtggcaaaccaagctcaacagctttccaaattctttgagtaatcaaacaggagaaaCCAATATGATCGTTTCTGacatcgaagctaacgccctcttagagctgtctcttatacacatctccg                                                                                                                                                                                                                                            >  1:638866‑M2/236‑279 (MQ=255)`  
                                                            `cacaccatgttcaaactgattaagaagttaggccaactgctggttcgtatgtacaacgtggaagccaagcgactgaacgatgaggctcgtaaagaggccacacagtcacgcgctctggcgattcgctccaacgaactggctgacagtgcatccactaaagttaccgaggctgcccgtgtggcaaaccaagctcaacagctttccaaattctttgagtaatcaaacagtagaaaTCAATATGATCGTTTCTGACATCGAAGCTAACGCCCTCTTAGAGAGCGTCACTAAGTTCCACTGCggg                                                                                                                                                                                                                                          <  1:223424‑M2/68‑1 (MQ=255)`  
                                                              `caccatgttcaaactgagtaagaagttaggccaactgctggttcgtatgtacaacgtggaagccaagcgactgaacgatgaggctcgtaaagaggccacacagtcacgcgctctgacgattcgctccaacgaactggctgacagtgcatccactaaagttaccgaggctgcccgtgtggcaaaccaagctcaacagctttccaaattctttgagtaatcaaacaggagaaaTCAATATGATCGTTTCTGACATCGAAGCTAACGCCCTCTTAGAGAGCGTCACTAAGTTCCACTGCGGGGt                                                                                                                                                                                                                                        <  1:822311‑M2/70‑1 (MQ=255)`  
                                                                   `tgttcaaactgattaagaagttaggccaactgctggttcgtatgtacaacgtggaagccaagcgattgaacgatgaggctcgtaaagaggccacacagtcacgcgctctggcgattcgctccaacgaactggctgacagtgcatccactaaagttaccgaggctgcccgtgtggcaaaccaagctcaacagctttccaaattctttgagtaatcaaacaggagaaaCCATTATGATCGTTTCTGACATCGAAGCTAACGCCCTCTTAGAGAGCGTCACTAAGTTCCACTGCGGGGTTATCt                                                                                                                                                                                                                                   <  1:534513‑M2/75‑1 (MQ=255)`  
                                                                    `gttcaaactgattaagaagttaggccaactgctggttcgtatgtacaacgtggaagccaagcgactgaacgatgaggctcgtaaagaggccacacagtcacgcgctctggcgattcgctccaacgaactggctgacagttcatccactaaagttaccgaggctgcccgtgtggcaaaccaagctcaacagctttccaaattctttgagtaatcaaacaggagaaaCCAATATGATCGTTTCTGACATCGAAGCTAACGCCCTCTTAGAGAGCGTAACTAATTTCCACTTCGGGTTTATCTa                                                                                                                                                                                                                                  >  1:622130‑M2/226‑301 (MQ=255)`  
                                                                        `aagctgattaagaagttaggccaactgctggttcgtatgtacaacgtggaagccaagcgactgaacgatgaggctcgtaaagaggccacacagtaacgcgctctggcgattcgctccaacgaactggctgacagtgcatccactaaagttaccgaggctgcccgtgtggcaaaccaagctcaacagctttccaaattctttgagtaatcaaacaggagaaaTCAATATGATCGTTTCTGACATCGAAGCTAACGCCCTCTTAGAGAGCGTCACAAAGTTCCACTGCGGGGTTATCTACGac                                                                                                                                                                                                                              <  1:541184‑M2/80‑1 (MQ=255)`  
                                                                        `aaactgattaagaagttaggccaactgctggttcgtatgtacaacgtggaagccaagcgactgaacgatgaggctcgtaaagaggccacacagtcacgcgctctggcgattcgctcaaacgaactggctgacagtgcatccactaaagttaccgaggctgcccgtgtggcaaaccaagctcaacagctttccaaattctttgagtaatcaaacaggagaaaTCAATATGATCGTTTCTGACATCGAAGCTAACGCCCTCTTAGAGAGCGTCACTAAGTTCCACTGCGGGGTTATCTACGac                                                                                                                                                                                                                              <  1:476164‑M2/80‑1 (MQ=255)`  
                                                                        `aaaatgattaagaagttaggcaaactgctggttcgaatgtacaacgtgacagccaagcgactgaacgatgaggctcgtaaagaggccacacagtcacgcgctctggcgattcgctccaacgaactggctgacagtgcatccactaaagttaccgaggctgcccgtgtggcaaaccaagctcaacagctttccaaattctttgagtaatcaaacaggagaaaTCAATATGATCGTTTCTGACATCGAAGCTAACGCCCTCTTAGAGAGCGTCACTAAGTTCCACTGCGGGGTTATCTACGac                                                                                                                                                                                                                              <  1:216953‑M2/80‑1 (MQ=255)`  
                                                                             `gattaagaagttaggccaactgctggttcgtatgtacaacgtggaagccaagcgactgaacgatgaggctcgtaaagaggccacacagtcacgcgctctggcgattcgctccaacgaactggctgacagtgcatccactaaagttaccgaggctgcccgtgtggcaaaccaagctcaacagctttccaaattctttgagtaatcaaacaggagaaaTCAATATGATCGTTTCTGACATCGAAGCTAACGCCCTCTTAGAGAGCGTCACTAAGTTCCACTGCGGGGTTATCTACGACTACTc                                                                                                                                                                                                                         >  1:312871‑M2/217‑301 (MQ=255)`  
                                                                                   `agacagaggccaactgctggttcgtatgtacaacgtggaagccaagcgactgaacgatgaggctcgtaaagaggccacacagtcacgcgctctggcgattcgctccaacgaactggctgacagtgcatccactaaagttaccgaggctgcccgtgtggcaaaccaagctcaacagctttccaaattctttgagtaatcaaacaggagaaaTCAATATGATCGTTTCTGACATCGAAGCTAACGCCCTCTTAGAGAGCGTCACTAAGTTCCACTGCGGGGTTATCTACGACTACTCCACCGc                                                                                                                                                                                                                   <  1:705379‑M2/91‑1 (MQ=255)`  
                                                                                   `aaagttaggccaactgctggttcgtatgtacaacgtggaagccaagcgactgaacgatgaggctcgtaaagaggccacacagtcacgcgctctggcgattcgctccaacgaactggctgacagtgcatccactaaagttaccgaggctgcccgtgtggcaaaccaagctcaacagctttccaaattctttgagtaatcaaacaggagaaaTCAATATGATCGTTTCTGACATCGAAGCTAACGCCCTCTTAGAGAGCGTCACTAAGTTCCACTGCGGGGTTATCTACGACTACTCCACCGc                                                                                                                                                                                                                   <  1:380660‑M2/91‑1 (MQ=255)`  
                                                                                      `gttaggccaactgctggttcgtatgtacaacgtggaagccaagcgactgaacgatgaggctcgtaaagaggccacacagtcacgcgctctggcgattcgctccaacgaactggctgacagtgcatccactaaagttaccgaggctgcccgtgtggcaaaccaagctcaacagctttccaaattctttgagtaatcaaacaggagaaaTCAATATGATCGTTTCTGACATCGAAGCTAACGCCCTCTTAGAGAGCGTCACTAAGTTCCACTGCGGGGTTATCTACGACTACTCCAccgcctg                                                                                                                                                                                                                >  1:735904‑M2/208‑298 (MQ=255)`  
                                                                                       `cagggccaactgctggttcgtatgtacaacgtggaagccaagcgactgaacgatgaggctcgtaaagaggccacacagtcacgcgctctggcgattcgctccaacgaactggctgacagtgcatccactaaagttaccgaggctgcccgtgtggcaaaccaagctcaacagctttccaaattctttgagtaatcaaacaggagaaaCCAATATGATCGTTTCTGACATCGAAGCTAACGCCCTCTTAGAGAGCGTCACTAAGTTCCACTGCGGGGTTATCTACGACTACTCCACCGCTGAg                                                                                                                                                                                                               <  1:778165‑M2/95‑1 (MQ=255)`  
                                                                                          `ggccaactgctggttcgtatgtacaacgtggaagccaagcgactgaacgatgaggctcgtaaagaggccacacagtcacgcgctctggcgattcgctccaacgaactggctgacagtgcatccactaaagttaccgaggctgcccgtgtggcaaaccaagctcaacagctttccaaattctttgagtaatcaaacaggagaaaTCAATATGATCGTTTCTGACATCGAAGCTAACGCCCTCTTAGAGAGCGTCACTAAGTTCCACTGCGGGGTTATCTACGACTACTCCACCGCTGAGTAc                                                                                                                                                                                                            <  1:338915‑M2/98‑1 (MQ=255)`  
                                                                                             `caactgctgtttcgtatgtacaacgtggaagccaagcgactgaacgatgaggctcgtaaagaggccacacagtcacgcgctctggcgattcgctctaacgaactggctgacagtgcatccactaaagttaccgaggctgcccgtgtggcaaaccaagctcaacagctttccaaattctttgagtaatcaaacaggagaaaCCATTATGATCGTTTCTGACATCGAAGCTAACGCCCTCTTAGAGAGCGTCACTAAGTTCCACTGCGGGGTTATCTACGACTACTCCACCGCTGAGTACGTa                                                                                                                                                                                                         <  1:59424‑M2/101‑1 (MQ=255)`  
                                                                                             `aaactactggttcgaatgtacaacgtgaaagccaagcgactgaacgatgaggctcgtaaagaggccacacagtcacgcgctcttgcgattcgccccaacggactggctgacagtgcatccactaaagttaccgaggctgcccgtggggcaaaccaagctcaacagctttccaaattctttgagtaatcaatcaggagaaaACAATATGATCGTTTCTGACATCGAAGCTAACGCCCTCTTAGAGAGCGTCACTAAGTTCCACTGCGGGGTTATCTACGACTACTCCACCGCTGAGTACGTa                                                                                                                                                                                                         <  1:63056‑M2/101‑1 (MQ=255)`  
                                                                                               `actgctggttcgtatgtacaacgtggaagccaagcgactgaacgatgaggctcgtaaagaggccacacagtcacgcgctctggcgattcgctccaacgaactggctgacagtgcatccactaaagttaccgaggctgcccgtgtggcaaaccaagctcaacagttttccaaattctttgagtaatcaaacaggagaaaTCAATATGATCGTTTCTGACATCGAAGCTAACGCCCTCTTAGAGAGCGTCACTAAGTTCCACTGCGGGGTTATCTACGACTACTCCACCGCTGAGTACGTAAg                                                                                                                                                                                                       <  1:332460‑M2/103‑1 (MQ=255)`  
                                                                                                  `gctggttcgtatgtacaacgtggaagccaagcgactgaacgatgaggctcgtaaagaggccacacagtcacgcgctctggcgattcgctccaacgaactggctgacagtgcatccactaaagttaccgaggctgcccgtgtggcaaaccaagctcaacaactttccaaattctttgagtaatcaaacaggagaaaTCAATATGATCGTTTCTGACATCGAAGCTAACGCCCTCTTAGAGAGCGTCACTAAGTTCCACTGCGGGGTTATCTACGACTACTCCACCGCTGAGTACGTAAGCTa                                                                                                                                                                                                    >  1:236347‑M2/196‑301 (MQ=255)`  
                                                                                                   `aagagacagatgtacaacgtggaagccaagcgactgaacgatgaggctcgtaaagaggccacacagtcacgcgctctggcgattcgctccaacgaactggctgacagtgcatccactaaagttaccgaggctgcccgtgtggcaaaccaagctcaacagctttccaaattctttgagtaatcaaacaggagaaaTCAATATGATCGTTTCTGACATCGAAGCTAACGCCCTCTTAGAGAGCGTCACTAAGTTCCACTGCGGGGTTATCTACGACTACTCCACCGATGAGTACGTAAGCTAc                                                                                                                                                                                                   <  1:319888‑M2/107‑1 (MQ=255)`  
                                                                                                         `cgtatgtacaacgtggaagccaagcgactgaacgatgaggctcgtaaagaggccacacagtcacgcgctctggcgattcgctccaacgaactggctgacagtgcatccactaaagttaccgaggctgcccgtgtggcaaaccaagctcaacagctttccaaattctttgagtaatcaaacaggagaaaCCATTATGATCGTTTCTGACATCGAAGCTAACGCCCTCTTAGAGAGCGTCACTAAGTTCCACTGCGGGGTTATCTACGACTACTCCACCGCTGAGTACGTAAGCTAACGTCCg                                                                                                                                                                                             >  1:305429‑M2/189‑301 (MQ=255)`  
                                                                                                          `cgtatgtacaacgtggaagccaagcgactgaacgatgaggctcgtaaagaggccacacagtcacgcgctctggcgattcgctccaacgaactggctgacagtgcatccactaaagttaccgaggctgcccgtgtggcaaaccaagctcaacagctttccaaattctttgagtaatcaataggagaaaTCAATATGATCGTTTCTGACATCGAAGCTAACGCCCTCTTAGAGAGCGTCACTAAGTTCCACTGCGGGGTTATCTACGACTACTCCACCGCTGAGTACGTAAGCTACCGTCCGa                                                                                                                                                                                            >  1:387422‑M2/188‑301 (MQ=255)`  
                                                                                                           `taggtaaaacgtggaagccaagcgactgaacgatgaggctcgtaaagaggccacacagtcacgcgctctggcgattcgctccaacgaactggctgacagtgcatccactaaagttaccgaggctgcccgtgtggcaaaccaagctcaacagctttccaaattctttgagtaatcaaacaggagaaaTCAATATGATCGTTTCTGACATCGAAGCTAACGCCCTCTTAGAGAGCGTCACTAAGTTCCACTGCGGGGTTATCTACGACTACTCCACCGCTGAGTACGTAAGCTACCGTCCGAg                                                                                                                                                                                           <  1:549726‑M2/115‑1 (MQ=255)`  
                                                                                                             `tgtacaacgtggaagccaagcgactgaacgatgaggctcgtaaagaggccacacagtcacgcgctctggcgattcgctccaacgaaatggctgacagtgcatccactaaagttaccgaggctgcccgtgtggcaaactaagctcaacagctttccaaattctttgagtaatcaaacaggagaaaTCAATATGATCGTTTCTGACATCGAAGCTAACGCCCTCTTAGAGAGCGTCACTAAGTTCCACTGCGGGGTTATCTACGACTACTCCACCGCTGAGTACGTAAGCTACCGTCCGAGTg                                                                                                                                                                                         <  1:259818‑M2/117‑1 (MQ=255)`  
                                                                                                              `gtacaacgtggaagccaagcgactgaacgatgaggctcgtaaagaggccacacagtcacgcgatctggcgattcgctccaacgaactggctgacagtgcatccactaaagttaccgagtctgcccgtgtggcaaaccaagctcaccagctttccaaattctttgagtagtcaaataggagaaaTCAATATGATCGTTACTGACATCGAAGCTAACTCCCTCTTAGACAGCGTCACTAAGTTCCACAGCGGGGGTATCTACGCCTACTCCACCGCTGAGTACGTGagatatcgtactatttt                                                                                                                                                                                        >  1:412931‑M2/184‑293 (MQ=255)`  
                                                                                                                    `cgtggaagccaagctactgaacgatgaggctcgtaaagaggccacacagtcacgcgctctggcgattcgctccaacgaactggctgacagtgcatccactaaagttaccgaggctgcccgtgtggcaaaccaagctcaacagctttccaaattctttgagtaatcaaacaggagaaaTCAATATGATCGTTTCTGACATCGAAGCTAACGCCATCTTAGAGAGCGTCACTAAGTTCCACTGCGGGGTTATCTACGACTACTCCACCGCTGAGTACGTAAGCTACCGTCCGAGTGACTTCgg                                                                                                                                                                                  >  1:616255‑M2/178‑301 (MQ=255)`  
                                                                                                                       `gtaagccaaaggactgaaagatgaggcccgtaaatatgccaaacagtcacacgctctggctattcgctccaacgaactggctgacagtgcatccactaaagttaccgaggcttcccgtgtggcaaaccaagctcaacagctttccaaattctttgagtaatctaacaggataaaTCAATATGATCGTTTCTGACATCGAAGCTAACGCCCTCTTAGAGAGCGTCACTAAGTTCCACTGCGGGGTTATCTACGACTACTCCACCGCTGAGTACGTAAGCTACCGTCCGAGTGACTTCGGTGc                                                                                                                                                                               <  1:108489‑M2/127‑1 (MQ=255)`  
                                                                                                                       `gagatgtgtataagagacagatgaggctcgtaaagaggccacacagtcacgcgctctggcgattcgctccaacgaactggctgacagtgcatccactaaagttaccgaggctgcccgtgtggcaaaccaagctcaacagctttccaaattctttgagtaatcaaacaggagaaaTCAATATGATCGTTTCTGACATCGAAGCTAACGCCCTCTTAGAGAGCGTCACTAAGTTCCACTGCGGGGTTATCTACGACTACTCCACCGCTGAGTACGTAAGCTACCTTCCGAGTGACTTCGGTGc                                                                                                                                                                               <  1:267654‑M2/127‑1 (MQ=255)`  
                                                                                                                        `gaagccaagcgactgaacgatgaggctcgtaaagaggccacacagtcacgcgctctggcgattcgctccaacgaactggctgacagtgcatccactaaagttaccgaggctgcccgtgtggcaaaccaagctcaacagctttccaaattctttgagtaatcaaacaggagaaaTCAATATGATCGTTTCTGACATCGAAGCTAACGCCCTCTTAGAGAGCGTCACTAAGTTCCACTGCGGGGTTATCTACGACTACTCCACCGCTGAGTACGTAAGCTACCGTCCGAGTGACTTCGGTGCg                                                                                                                                                                              >  1:274817‑M2/174‑301 (MQ=255)`  
                                                                                                                        `gaagccaagcgactgaacgatgaggctcgtaaagaggccacacagtcacgcgctctggcgattcgctccaacgaactggctgacagtgcatccactaaagttaccgaggctgcccgtgtggcaaaccaagctcaacagctttccaaattctttgagtaatcaaacaggagaaaCCATTATGATCGTTTCTGACATCGAAGCTAACGCCCTCTTAGAGAGCGTCACTAAGTTCCACTGCGGGGTTATCTACGACTACTCCACCGCTGAGTACGTAAGCTACCGTCCGAGTGACTTCGGTGCg                                                                                                                                                                              <  1:610472‑M2/128‑1 (MQ=255)`  
                                                                                                                              `caagcgactgaacgatgaggctcgtaaagaggccacacagtcacgcgctctggcgattcgctccaacgaactggctgacagtgcatccactaaagttaccgaggctgcccgtgtggcaaaccaagctcaacagctttccaaattctttgagtaatcaataggagaaaTCAATATGATCGTTTCTGACATCGAAGCTAACGCCCTCTTAGAGAGCGTCACTAAGTTCCACTGCGGGGTTATCTACGACTACTCCACCGCTGAGTACGTAAGCTACCGTCCGAGTGACttcggtgctgtctct                                                                                                                                                                        >  1:445819‑M2/168‑294 (MQ=255)`  
                                                                                                                                `gcgactgaacgatgaggctcgtaaagaggccacacagtcacgcgctctggcgattctctccaacgaactggctgacagtgcatccactaaagttaccgaggctgcccgtgtggcaaaccaagctcaacagctttccaaattctttgagtaatcaaacaggagtaaTCAATATGATCGTTTCTGACATCGAAGCTAACGCCCTCTTAGAGAGCGTCACTAAGTTCCACTGCGGGGTTATCTACGACTACTCCACCGCTGAGTACGTAAGCTACCGTCCGAGTGACTTCGGTGCGTATCtggg                                                                                                                                                                      >  1:520147‑M2/166‑300 (MQ=255)`  
                                                                                                                                `gcgactgaacgatgaggctcgtaaagaggccacacagtcacgcgctctggcgattcgctccaacgaactggctgacagtgcatccactaaagttaccgaggctgcccgtgtggcaaaccaagctcaacagctttccaaattctttgagtaatcaaacaggagaaaTCAATATGATCGTTTCTGACATCGAAGCTAACGCCCTCTTAGAGAGCGTCACTAAGTTCCACTGCGGGGTTATCTACGACTACTCCACCGCTGAGTACGTAAGCTACCGTCAGAGTGACTTCGGTGCGTATCTGGa                                                                                                                                                                      <  1:498209‑M2/136‑1 (MQ=255)`  
                                                                                                                                                       `aagagacagcccagtcacgcgctctggcgattcgctccaacgaactggctgacagtgcatccactaaagttaccgaggctgcccgtgtggcaaaccaagctcaacagctttccaaattctttgagtaatcaaccaggagaaaTCAATATGATCGTTTCTGACATCGAAGCTAACGCCCTCTTAGAGAGCGTCACTAAGTTCCACTGCGGGGTTATCTACTACTACTCCACCGCTGAGTACGTAAGCTACCGTCCGAGTGACTTCGGTGCGTATCTGGATGCGCTGGAAGCCGAGGTTGCAc                                                                                                                                               <  1:354180‑M2/159‑1 (MQ=255)`  
                                                                                                                                                         `gaggccacacagtcacgcgctctggcgattcgctccaacgaactggctgacagtgcatccactaaagttaccgaggctgcccgtgtggcaaaccaagctcaacagctttccaaattctttgagtaatcaaacaggagaaaTCAATATGATCGTTTCTGACATCGAAGCTAACGCCCTCTTAGAGAGCGTCACTAAGTTCCACTGCGGGGTTATCTACGACTACTCCACCGCTGAGTACGTAAGCTACCGTCCGAGTGACTTCGGTGCGTATCTGGATGCGCTGGAAGCCGAGGTTGCAcgg                                                                                                                                             >  1:92187‑M2/141‑300 (MQ=255)`  
                                                                                                                                                          `aggccacacagtcacgcgctctggcgattcgctccaacgaactggctgacagtgcatccactaaagttaccgaggctgcccgtgtggcaaaccaagctcaacagctttccaaattctttgagtaatcaaacaggagaaaTCAATATGATCGTTTCTGACATCGAAGCTAACGCCCTCTTAGAGAGCGTCACTAAGTTCCACTGCGGGGTTATCTACGACTACTCCACCGCTGAGTACGTAAGCTACCGTCCGAGTGACTTCGGTGCGTATCTGGATGCGCTGGAAGCCGAGGTTGCACGAg                                                                                                                                            >  1:506408‑M2/140‑301 (MQ=255)`  
                                                                                                                                                                  `cagtcacgcgctctggcgattcgctccaacgaactggctgacagtgcatccactaaagttaccgaggctgcccgtgtggcaaaccaagctcaacagctttccaaattctttgagtaatcaaacaggagaaaTCAATATGATCGTTTCTGACATCGAAGCTAACGCCCTCTTAGAGAGCGTCACTAAGTTCCACTGCGGGGTTATCTACGACTACTCCACCGCTGCGTACGTAAGCTACCGTCCGAGTGACTTCGGTGCGTATCTGGCTGCGCTGGAAGCCGAGGTTGCACGAGGCGGTCtt                                                                                                                                    >  1:661260‑M2/132‑301 (MQ=255)`  
                                                                                                                                                                    `gtcacgcgctctggcgattcgctccaacgaactggctgacagtgcatccactaaagttaccgaggctgcccgtgtggcaaaccaagctcaacagctttccaaattctttgagtaatcaaacaggagaaaTCAATATGATCGTTTCTGACATCGAAGCTAACGCCCTCTTAGAGAGCGTCACTAAGTTCCACTGCGGGGTTATCTACGACTACTCCACCGCTGAGTACGTAAGCTACCGTCCGAGTGACTTCGGTGCGTATCTGGATGCGCTGGAAGCCGAGGTTGCACGAGGCGGTCTTAt                                                                                                                                  >  1:685304‑M2/130‑301 (MQ=255)`  
                                                                                                                                                                      `cacgcgctctggcgattcgctccaacgaactggctgacagtgcatccactaaagttaccgaggctgcccgtgtggaaaaccaagctcaacagctttccaaattctttgagtaatcaaacaggagaaaTCAATATGATCGTTTCTGACATCGAAGCTAACGCCCTCTTAGAGAGCGTCACTAAGTTCCACTGCGGGGTTATCTACGACTACTCCACCGCTGAGTACGTAAGCTACCGTCCGAGTGACTTCGGTGCGTATCTGGATGCGCTGGAAGCCGAGGTTGCACGAGGCGGTCTTATtg                                                                                                                                <  1:527139‑M2/174‑1 (MQ=255)`  
                                                                                                                                                                      `agagacagctggcgattcgctccaacgaactggctgacagtgcatccactaaagttaccgaggctgcccgtgtggcaaaccaagctcaacagctttccaaattctttgagtaatcaaacaggagaaaTCAATATGATCGTTTCTGACATCGAAGCTAACGCCCTCTTAGAGAGCGTCACTAAGTTCCACTGCGGGGTTATCTACGACTACTACACCGCTGAGTACGTAAGCTACCGTCCGAGTGACTTCGGTGCGTATCTGGATGCGCTGGAAGCCGAGGTTGCACGAGGCGGTCTTATtg                                                                                                                                <  1:468962‑M2/174‑1 (MQ=255)`  
                                                                                                                                                                      `aacgcgctctggcgattcgatccaacgaactggctgacagtgcatccactaaagttaccgaggctgcccgtgtggcaaaacaagctcaacagctttccaaattctttgagtaatcaaacaggagaaaTCAATATGATCGTTTCTGACATCGAAGCTAACGCCATCTTAGAGAGCGTCACTAAGTTCCACTGCGGGGTTATCTACGACTACTCCACCGCTGAGTACGTAAGCTACCGTCCGAGTGACTTCGGTGCGTATCTGGATGCGCTGGAAGCCGAGGTTGCACGAGGCGGTCTTATtg                                                                                                                                <  1:578281‑M2/174‑1 (MQ=255)`  
                                                                                                                                                                           `gctctggcgattcgctccaacgaactggctgacagtgcatccactaaagttaccgaggctgcccgtgtggcaaaccaagctaaacagctttccaaattctttgagtaatcaaacaggagaaaTCAATATGATCGTTTCTGACATCGAAGCTAACGCCCTCTTAGAGAGCGTCACTAAGTTCCACTGCGGGGTTATCTACGACTACTCCACCGCTGAGTACGTAAGCTACCGTCCGAGTGACTTCGGTGCGTATCTGGATGCGCTGGAAGCCGAGGTTGCACGAGGCGGTCTTATTGTGTTc                                                                                                                           <  1:51314‑M2/179‑1 (MQ=255)`  
                                                                                                                                                                            `ctctggcgattcgctccaacgaactggctgacagtgcatccactaaagttaccgaggctgcccgtgtggcaaaccaagctcaacagctttccaaattctttgagtaatcaaacaggagaaaTCAATATGATCGTTTCTGACATCGAAGCTAACGCCCTCTTAGAGAGCGTCCCTAAGTTTCACTGCGGGGTTATCTACGACTACTCCACCGCTGCGTACGTAAGCTACCGTCCGAGTGCCTTCGGTGCGTCTCTGGATGCGTTGGAAGCCGAGGTTGCACGAGGCGGTCTTATTGTGTTcc                                                                                                                          >  1:201626‑M2/122‑301 (MQ=255)`  
                                                                                                                                                                                        `gctccaacgaactggctgacagtgcatccactaaagttaccgaggctgcccgtgtggcaagccaagctcaacagctttccaaattctttgagtaatcaaacaggagaaaTCAATATGATCGTTTCTGACATCGAAGCTAACGCCCTCTTAGAGAGCGTCACTAAGTTCCACTGCGGGGTTATCTACGACTACTCCACCGCTGAGTACGTAAGCTACCGTCCGAGTGACTTCGGTGCGTATCTGGATGCGCTGGAAGCCGAGGTTGCACGAGGCGGTCTTATTGTGTTCAACAACGGTcaca                                                                                                              >  1:371307‑M2/110‑301 (MQ=255)`  
                                                                                                                                                                                        `gctccaacgaactggctgacagtgcatccactaaagttaccgaggctgcccgtgtggcaaaccaagctcaacagctttccaaattctttgagtaatcaaacaggagaaaTCAATATGATCGTTTCTGACATCGAAGCTAACGCCCTCTTAGAGAGCGTCACTAAGTTCCACTGCGGGGTTATCTACGACTACTCCACCGCTGAGTACGTAAGCTACCGTCCGAGTGACTTCGATGCGTATCTGGATGCGCTGGAAGCCGAGGTtacacgaggcggtcttattgctgtctctcatacacatt                                                                                                              >  1:173976‑M2/110‑283 (MQ=255)`  
                                                                                                                                                                                                                  `tccactaaagttaccgaggctgcccgtgtggcaaaccaagctcaacagctttccaaattctttgagtaatcaaacaggagaaaTCAATATGATCGTTTCTGACATCGAAGCTAACGCCCTCTTAGAGAGCGTCACTAAGTTCCACTGCGGGGTTATCTACGACTACTCCACCGCTGAGTACGTAAGCTACCGTCCGAGTGACTTCGGTGCGTATCTGGATGCGCTGGAAGCCGAGGTTGCACGAGGCGGTCTTATTGTGTTCCACAACGGTCACAAGTATGACGTTCCTGCATTGACCaaa                                                                                    >  1:460893‑M2/84‑301 (MQ=255)`  
                                                                                                                                                                                                                   `ccactaaagttaccgaggctgcccgtgtggcaaaccaagctcaacagctttccaaattctttgagtaatcaaacaggagaaaTCAATATGATCGTTTCTGACATCGAAGCTAACGCCCTCTTAGAGAGCGTCACTAAGTTCCACTGCGGGGTTATCTACGACTACTCCACCGCTGAGTACGTAAGCTACCGTCCGAGTGACTTCGGTGCGTATCTGGATGCGCTGGAAGCCGAGGTTGCACGAGGCGGTCTTATTGTGTTCCACAACGGTCACAAGTATGACGTTCCTGCATTGACcaaaa                                                                                   >  1:808634‑M2/83‑300 (MQ=255)`  
                                                                                                                                                                                                                          `agttaccgaggctgcccgtgtggcaaaccaagctcaacagctttccaaattctttgagtaatcaaacaggagaaaTCAATATGATCGTTTCTGACATCGAAGCTAACGCCCTCTTAGAGAGCGTCACTAAGTTCCACTGCGGGGTTATCTACGACTACTCCACCGCTGAGTACGTAAGCTACCGTCCGAGTGACTTCGGTGCGTATCTGGATGCGCTGGAAGCCGAGGTTGCACGAGGCGGTCTTATTGTGTTCCACAACGGTCACAAGTATGACGTTCCTGAATTGACCAAACTGGCaaa                                                                            >  1:83701‑M2/76‑301 (MQ=255)`  
                                                                                                                                                                                                                          `agttaccgaggctgcccgtgtggcaaaccaagctcaacagctttccaaattctttgagtaatcaaacaggagaaaTCAATATGATCGTTTCTGACATCGAAGCTAACGCCCTCTTAGAGAGCGTCACTAAGTTCCACTGCGGGGTTATCTACGACTACTCCACCGATGAGTACGTAAGCTACCGTCCGAGTGACTTCGGTGCGTATCTGGATGCGCTGGAAGCCGAGGTTGCACGAGGCGGTCTTATTGTGTTCCACAACGGTCACAAGTATGACGTTCCTGCATTGACCAAACTGGCaaa                                                                            >  1:182057‑M2/76‑301 (MQ=255)`  
                                                                                                                                                                                                                                  `gggctgcccgtgtggcaaaccaagctcaacagctttccaaattctttgagtaatcaaacaggagaaaTCAATATGATCGTTTCTGACATCGAAGCTAATGCCCTCTTAGAGAGCGTCACTAAGTTCCACTGCGGGGTTATCTACGACTACTCCACCGCTGAGTACGTAAGCTACCGTCCGAGTGACTTCGGTGCGTATCTGGATGCGCTGGAAGCCGAGGTTGCACGAGGCGGTCTTATTGTGTTCCACAACGGTCACAAGTATGACGTTCCTGCATTGACCAAACTGGCAAAGTTGCAAt                                                                    <  1:429941‑M2/234‑1 (MQ=255)`  
                                                                                                                                                                                                                                            `tgtggcaaaccaagctcaacagctttccaaattctttgagtaatcaaacaggagaaaTCAATATGATCGTTTCTGACATCGAAGCTAACGCCCTCTTAGAGAGCGTCACTAAGTTCCACTGCGGGGTTATCTACGACTACTCCACCGCTGAGTACGTAAGCTACCGTCCGAGTGACTTCGGTGCGTATCTGGATGCGCTGGAAGCCGAGGTTGCACGAGGCGGTCTTATTGTGTTCCACAACGGTCACAAGTATGACGTTCCTGCATTGACCAAACTGGCAAAGTTGCAATTGAACCgaga                                                          <  1:58577‑M2/244‑1 (MQ=255)`  
                                                                                                                                                                                                                                             `gtggcaaaccaagctcaacagctttccaaattctttgagtaatcaaacaggagaaaTCAATATGATCGTTTCTGACATCGAAGCTAACGCCCTCTTAGAGAGCGTCACTAAGTTCCACTGCGGGGTTATCTACGACTACTCCACCGCTGAGTACGTAAGCTACCGTCCGAGTGACTTCGGTGCGTATCTGGATGCGCTGGAAGCCGAGGTTGCACGAGGCGGTCTTATTGTGTTCCACAACGGTCACAAGTATGACGTTCCTGCATTGACCAAACTGGCAAAGTTGCAATTGAAccgaggg                                                         >  1:490232‑M2/57‑299 (MQ=255)`  
                                                                                                                                                                                                                                                   `aaccaagctcaacagctttccaaatacgttgagtaatccaacaggagaaaTCAATATGATCGTTTCTGACATCGAAGATAACGCCCTCTAAGAGAGCGTCACTAAGGTCCACTGCGGGGTTATCTAAGACTACTCCACCGCTGAGTACGTAAGCTACCGTCCGAGTGACTTCGGTGCGGATCTGGATGCGATGGAAGCCGAGGTTGCACGAGGCGGTCTTATTGTGTTCCACAACGGTCACAAGTATGACGTTCCTGCATTGACCAAACTGGCAAAGTTGCAATTGAACCGAGAGTTCCAc                                                   <  1:18933‑M2/251‑1 (MQ=255)`  
                                                                                                                                                                                                                                                       `aagctcaacagctttccaaattctttgagtaatcaaacaggcgaaaTCAATATGATCGTTTCTGACATCGACGCTAACGCCCTCTTAGAGAGCGTCACTAAGTTCCACTGCGGGGTTATCTACGACTACTCCACCGCTGAGTACGTAAGCTACCGTCCGAGTGACTTCGGTGCGTATCTGGATGCGCTGGAAGCCGAGGTTGCACGAGGCGGTCTTATTGTGTTCCACAACGGTCACAAGTATGACGTTCCTGCATTGACCAAACTGGCAAAGTTGCAATTTAACCGAGAGTTCCACCTTc                                               >  1:252846‑M2/47‑301 (MQ=255)`  
                                                                                                                                                                                                                                                         `gctcaacagctttccaaattctttgagtaatcaaacaggagaaaTCAATATGATCGTTTCTGACATCGAAGCTAACGCCCTCTTAGAGAGCGTCACTAAGTTCCACTGCTGGGTTATCTACGACTACTCCACCGCTGAGTACGTAAGCTACCGTCCGAGTGACTTCGGTGCGTATCTGGATGCGCTGGAAGCCGAGGTTGCACGAGGCGGTCTTATTGTGTTCCACAACGGTCACAAGTATGACGTTCCTGCATTGACCAAACTGGCAAAGTTGCAATTGAACCGAGAGTTCcacctctgt                                             >  1:127776‑M2/45‑297 (MQ=255)`  
                                                                                                                                                                                                                                                         `gctcaacagctttccaaaatctttgaataatcaaacaggagaaaTCAAGATGATCGTTTCTGACATCGAAGCTAACGCCCTCTTAGAGAGCGTCACTAAGTTCCACTGCGGGGTTATCTACGGCTACTCCACCGCTGAGTACGTAAGCTACCGTCCGAGTGACTTCGGTGCGTATCTGGATGCGCTGGAAGCCGAGGTTGCACGAGGCGGTCTTATTGTGTTCCACAACGGTCACAAGTATGACGTTCCTGCATTGACCAAACTGGCAAAGTTGCAATTGAACCGAGAGTTCCACCTTCCt                                             <  1:195045‑M2/257‑1 (MQ=255)`  
                                                                                                                                                                                                                                                            `caacagctttccaaattctttgagtaatcaaataggagaaaTCAATATGATCGTTTCTGACATCGAAGCTAACGCCCTCTTAGAGAGCGTCACTAAGTTCCACTGCGGGGTTATCTACGACTACTCCACCGCTGAGTACGTAAGCTACCGTCCGAGTGACTTCGGTGCGTATCTGGATGCACTGGAAGCCGAGGTTGCACGAGGCGGTCTTATTGTGTTCCACAACGGTCACAAGTATGACGTTCCTGCATTGACCAAACTGGCAAAGTTGCAATTGAACCGAGAGTTCCACCTTCCTCGt                                          >  1:290174‑M2/42‑301 (MQ=255)`  
                                                                                                                                                                                                                                                                 `gctttccaaattctttgagtaatcaaacaggagaaaTCAATATGATCGTTTCTGACATCGAAGCTAACGCCCTCTTAGAGAGCGTCACTAAGTTCCACTGCGGGGTTATCTACGACTACTCCACAGCTGAGTACGTAAGCTACCGTCCGAGTGACTTCGGTGCGTATCTGGATGCGCTGGAAGCCGAGGTTGCACGAGGCGGTCTTATTGTGTTCCACAACGGTCACAAGTATGACGTTCCTGCATTGACCAAACTGGCAAAGTTGCAATTGAACCGAGAGttccaccttccctgtctctt                                     >  1:210698‑M2/37‑292 (MQ=255)`  
                                                                                                                                                                                                                                                                    `gagacagttctttgagtaatcaaacaggagaaaTCAATATGATCGTTTCTGACATCGAAGCTAACGCCCTCTTAGAGAGCGTCACTAAGTTCCACTGCGGGGTTATCTACGACTACTCCACCGCTGAGTACGTAAGCTACCGTCCGAGTGACTTCGGTGCGTATCTGGATGCGCTGGAAGCCGAGGTTGCACGAGGCGGTCTTATTGTGTTCCACAACGGTCACAAGTATGACGTTCCTGCATTGACCAAACTGGCAAAGTTGCAATTGAACCGAGAGTTCCACCTTCCTCGTGAGAACTg                                  <  1:29735‑M2/268‑1 (MQ=255)`  
                                                                                                                                                                                                                                                                       `caaattctttgagtaatcaaacaggagaaaTCAATATGATCGTTTCTGACATCGAAGCTAACGCCCTCTTAGAGAGCGTCACTAAGTTCCACTGCGGGGTTATCTACGACTACTCCACCGCTGAGTACGTAAGCTACCGTCCGAGTGACTTCGGTGCGTATCTGGATGCGCTGGAAGCCGAGGTTGCACGAGGCGGTCTTATTGTGTTCCACAACGGTCACAAGTATGACGTTCCTGCATTGACCAAACTGGCAAAGTTGCAATTGAACCGAGAGTTCCACCTTCCTCGTGAGAACTGTAt                               <  1:691771‑M2/271‑1 (MQ=255)`  
                                                                                                                                                                                                                                                                           `ttctttgagtaatcaaacaggagaaaCCATTATGATCGTTTCTGACATCGAAGCTAACGCCCTCTTAGAGAGCGTCACTAAGTTCCACTGCGGGGTTATCTACGACTACTCCACCGCTGAGTACGTAAGCTACCGTCCGAGTGACTTCGGTGCGTATCTGGATGCGCTGGAAGCCGAGGTTGCACGAGGCGGTCTTATTGTGTTCCACAACGGTCACAAGTATGACGTTCCTGCATTGACCAAACTGGCAAAGTTGCAATTGAACCTAGAGTTCCACCTTCCTCGTGAGAACTGTATTGac                           >  1:453341‑M2/27‑301 (MQ=255)`  
                                                                                                                                                                                                                                                                            `ttctttgagtaatcaataggagaaaTCAATATGATCGTTTCTGACATCGAAGCTAACGCCCTCTTAGAGAGCGTCACTAAGTTCCACTGCGGGGTTATCTACGACTACTCCACCGCTGAGTACGTAAGCTACCGTCCGAGTGACTTCGGTGCGTATCTGGATGCGCTGGAAGCCGAGGTTGCACGAGGCGGTCTTATTGTGTTCCACAACGGTCACTAGTATGACGTTCCTGCATTGACCAAACTGGCAAAGTTGCAATTGAACCTAGAGTTCCACCTTCCTCGTGAGAACTGTATTGaca                          >  1:325176‑M2/26‑301 (MQ=255)`  
                                                                                                                                                                                                                                                                              `tttgagtaatcaaacaggagaaaTCAATATGATCGTTTCTGACATCGAAGCTAACGCCCTCTTAGAGAGCGTCACTAAGTTCCACTGCGGGGTTATCTACGACTACTCCACCGCTGAGTACGTAAGCTACCGTCCGAGTGACTTCGGTGCGTATCTGGATGCGCTGGAAGCCGAGGTTGCACGAGGCGGTCTTATTGTGTTCCACAACGGTCACAAGTATGACGTTCCTGCATTGACCAAACTGGCAAAGTTGCAATTGAACCGAGAGTTCCACCTTCCTCGTGAGAACTGTATTGACAcc                        <  1:114490‑M2/278‑1 (MQ=255)`  
                                                                                                                                                                                                                                                                                  `agtaaacaaacaggagaaaTCAATATGATCGTTTCTGACATCGAAGCTAACGCCCTCTTAGAGAGCGTCACTAAGTTCCACTGCGGGGTTATCTACGACTACTCCACCGCTGAGTACGTAAGCTACCGTCCGAGTGACTTCGGTGCGTATCTGGATGCGCTGGAAGCCGAGGTTGCACGAGGCGGTCTTATTGTGTTCCACAACGGTCACAAGTATGACGTTCCTGCATTGACCAAACTGGCAAAGTTGCAATTGAACCGAGAGTTCCACCTTCCTCGTGAGAACTGTATTGACACCCTtg                    <  1:720901‑M2/282‑1 (MQ=255)`  
                                                                                                                                                                                                                                                                                              `ggagaaaCCATTATGATCGTTTCTGACATCGAAGCTAACGCCCTCTTAGAGAGCGTCACTAAGTTCCACTGCGGGGTTATCTACGACTACTCCACCGATGAGTACGTAAGCTACCGTCCGAGTGACTTCGGTGCGTATCTGGATGCGATGGAAGCCGAGGTTGCACGAGGCGGTCTTATTGTGTTCCACAACGGTCACAAGTATGACGTTCCTGCATTGACCAAACTGGCAAAGTTGCAATTGAACCGAGAGTTCCACCTTCCTCGTGAGAACTGTATTGACACCCTTGTGTTGtcactgt        >  1:8156‑M2/8‑298 (MQ=255)`  
                                                                                                                                                                                                                                                                                                    `aTCAAGATGATCGTTTCTGACATCGAAGCTAACGCCCTCTTAGAGAGCGTCACTAAGTTCCACTGCGGGGTTATCTACGACTACTCCACCGCTGAGTACGTAAGCTACCGTCCGAGTGACTTCGGTGCGTATCTGGATGCGCTGGAAGCCGAGGTTGCACGAGGCGGTCTTATTGTGTTCCACAACGGTCACAAGTATGACGTTCCTGCATTGACCAAACTGGCAAAGTTGCAATTGAACCGAGAGTTCCACCTTCCTCGTGAGAACTGTATTGACACCCTTGTGTTGTCACGTTTGATTc  <  1:800513‑M2/300‑1 (MQ=255)`  

                                                                                                                                                                                                                                                                                                     `|`                                                                                                                                                                                                                                                                                                             
`AAGACAGACTGGCTGGAAGAAACCAGAAGTTACCAAGCTGAGTCATAAGGTGTTCGATAAGGACACTATGACCCACATCAAGGCTGGTGATTGGGTTAAGGTTGACATGGGAGTTGTTGGTGGATACGGCTACGTCCGCTCAGTTAGTGGCAAATATGCACAAGTGTCATACATCACAGGTGTTACTCCACGCGGTGCAATCGTTGCCGATAAGACCAACATGATTCACACAGGTTTCTTGACAGTTGTTTCATATGAAGAGATTGTTAAGTCACGATAATCAATAGGAGAAATCAATATGATCGTTTCTGACATCGAAGCTAACGCCCTCTTAGAGAGCGTCACTAAGTTCCACTGCGGGGTTATCTACGACTACTCCACCGCTGAGTACGTAAGCTACCGTCCGAGTGACTTCGGTGCGTATCTGGATGCGCTGGAAGCCGAGGTTGCACGAGGCGGTCTTATTGTGTTCCACAACGGTCACAAGTATGACGTTCCTGCATTGACCAAACTGGCAAAGTTGCAATTGAACCGAGAGTTCCACCTTCCTCGTGAGAACTGTATTGACACCCTTGTGTTGTCACGTTTGATTC  >  NC_001604.1/14055‑14647`

  

Alignment Legend

Aligned base mismatch/match (shaded by quality score): `ATCG/ATCG` `< 3 ≤` `ATCG/ATCG` `< 13 ≤` `ATCG/ATCG` `< 26 ≤` `ATCG/ATCG` `< 37 ≤` `ATCG/ATCG` `< 38 ≤` `ATCG/ATCG`

Unaligned base: `atcg`    Masked matching base: `atcg`    Alignment gap: `‑`    Deleted base: `‑`
